# Different on the abundance of *Pampus argenteus* in Persian Gulf exceeding in variety comparing to Gulf of Oman

**DOI:** 10.1080/23802359.2017.1357434

**Published:** 2017-09-26

**Authors:** Atena Sheibaninia, Parisa Nejatkhah Manavi, Massoud Houshmand

**Affiliations:** a Department of Marine Biology, Faculty of Marine Science and Technology, Islamic Azad University, Tehran, Iran;; b Department of Medical Genetic, National Institute of Genetic Engineering and Biotechnology, Tehran, Iran

**Keywords:** *Pampus argenteus*, cytochrome b, D-loop, Persian Gulf, Gulf of Oman

## Abstract

Silver pomfret fish (Stromateidae) is a family of Perciformes order. One species of this family is *Pampus argenteus* known as silver pomfret and lives in different parts of the Persian Gulf and Gulf of Oman. Therefore, the genetic relationship between its populations is so important. In this study, eight samples of *Pampus argenteus* were collected from four locations involved: Emam Khomeyni, Bushehr and Bandar Abbas in Persian Gulf and Chabahar in Gulf of Oman. Cytochrome b gene and D-loop region of mitochondrial DNA on these samples are analyzed. These genes are carried out using PCR with specific mtDNA primers and sequenced. Then, the evolutionary history was inferred by using the maximum-likelihood method. Evolutionary analyses were conducted in MEGA7. According to the phylogenetic trees, with translocations in Persian Gulf to Gulf of Oman, similarity decreases slowly. Chabahar1 sample in phylogenetic tree of cytochrome b is phylogenetically near China Sea samples. The reason may be migration from ocean to gulf throughout the centuries and Chabahar is in Gulf of Oman and locationally near the Arabian Sea and Indian Ocean. In D-loop region, results are similar to cytochrome b excepting one sample (Emam Khomeyni1). D-Loop in samples shows modification more than cytochrome b for the reason absence of protein product.

## Introduction

There is silver pomfret in different parts of Persian Gulf and Gulf of Oman. Silver pomfret fish (Stromateidae) is a family of fish Perciformes order. This fish is bilateral body and its caudal fin is short. It is not visible any gill plate. Maximum length is 60 cm.

Silver pomfret is known *Pampus argenteus*. Feeding value of silver pomfret fish is high and this fish has many clients in the world. Other names for this fish are silver butterfish, white pomfret and Zobeidi. The species exists in the Persian Gulf and Gulf of Oman, Arabian Sea, Indian Ocean, Atlantic Ocean, Bay of Bengal, East and South China Sea, Yellow Sea, Indonesia Sea, Mediterranean and Pacific Ocean nowadays. These fish nourish zooplankton catch by gillnets (Li et al. 2014).

Mitochondrial DNA in vertebrates is protected very low and show high evolutionary rate (Aboim 2005). Molecular studies of the silver pomfret fish in Iran have been done, but the results are not phylogenetic study. In other country, silver pomfret fish is considered as follows: Li et al. 2014 used molecular techniques to sequence the entire mitochondrial genome which is 16,544 bp. The origin of *Pampus argenteus* may vary. Imbrie et al. 1992 suggest that the end of the Pleistocene period had significant evidences of genetic populations of silver pomfret.

In this study, sequences of eight samples of *Pampus argenteus* in four areas were considered and compared with Chinese sequences for appointment of their similarity and difference between them and evolutionary history was distinguished.

## Materials and methods

**Figure 1.**
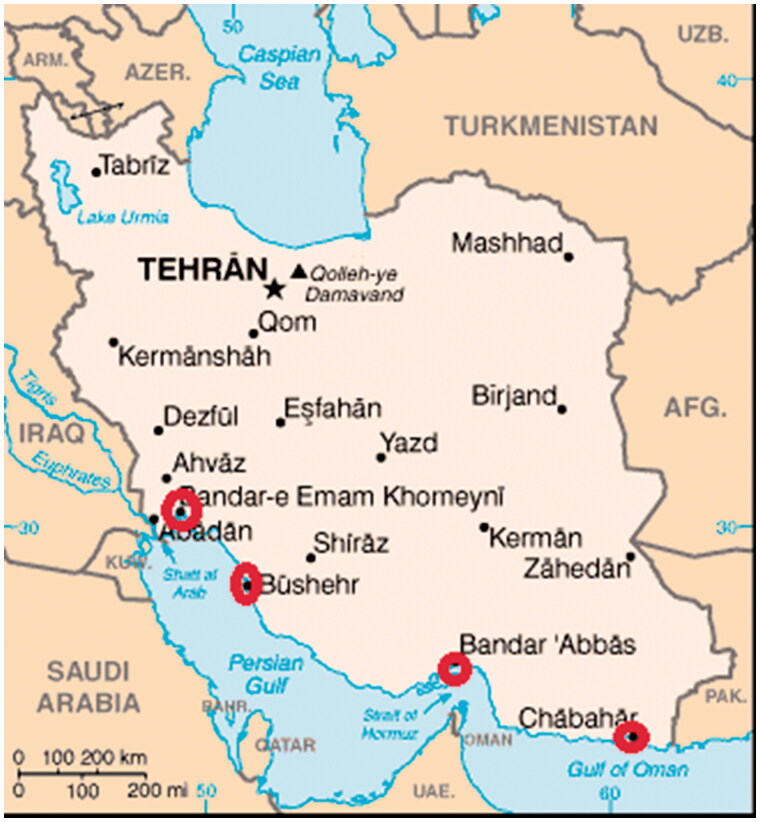
The geographical locations and sampling areas: Emam Khomeyni, Bushehr and Bandar Abbas in Persian Gulf and Chabahar in Gulf of Oman.

Eight samples of *Pampus argenteus* were collected in May 2013 from four locations involved: Emam Khomeyni, Bushehr and Bandar Abbas in Persian Gulf and Chabahar in Gulf of Oman. Two samples were brought in each area. The geographical locations and sampling areas are shown in Figure 1.

Genomic DNA was extracted from a piece of muscle tissue of all samples, using a QIAmap DNA Micro Kit (Qiagen #56304). For DNA crystallization, chloroform, Nacl_2_ and ethanol 100% were added and finally DNA was rinsed with ethanol 70%. There were no submitted sequences of *Pampus argenteus* cytochrome b and D-loop in the GenBank; therefore, we used the universal primers (Sun et al. 2012) (Cyt b forward primer: 5′-GACTTCAAAAACCACCGTT-3′; Cyt b reverse primer: 5′-CTCCGATCTCCGGATTACAAGAC-3′; D-loop forward primer: 5′-TGTAAACCGGACAAGTCGGGAG-3′; D-loop reverse primer: 5′-GTGGCTAGGAAGGTGTCAT-3′). All samples were sequenced on an ABI 3700 sequencer (Kosar Company, Tehran, Iran) and analyzed in MEGA7 (Kumar et al. 2016).

## Results

Finch TV software was used to analyze the DNA sequences. Sequences send to GenBank BankIt (D-loop region Submission ID: 1987916 and cytochrome b gene Submission ID: 1987966). A phylogenetic tree was drawn based on comparing cytochrome b and D-loop sequences of *Pampus argenteus* in eight samples and six sequences of *Pampus* that were obtained in the GenBank (https://www.ncbi.nlm.nih.gov/): (1) *Pampus-spLY-2009*, (2) *Pampus argenteus*, (3) *Pampus chinensis*, (4) *Pampus echinogaster*, (5) *Pampus punctatissimus* and (6) *Peprilus triacanthus*. All these species were located on China Sea.

The evolutionary history was inferred by using the maximum-likelihood method base on the Tamura–Nei model (Tamura and Nei 1993). Initial tree(s) for the heuristic search were obtained automatically by applying neighbour-joining and BioNJ algorithm to a matrix of pairwise distance estimated using the maximum composite likelihood (MCL) approach and then selecting the topology with superior log-likelihood value. The analysis involved 14 nucleotide sequences. All positions containing gaps and missing data were eliminated. Evolutionary analyses were conducted in MEGA7 (Kumar et al. 2016).

**Figure 2.**
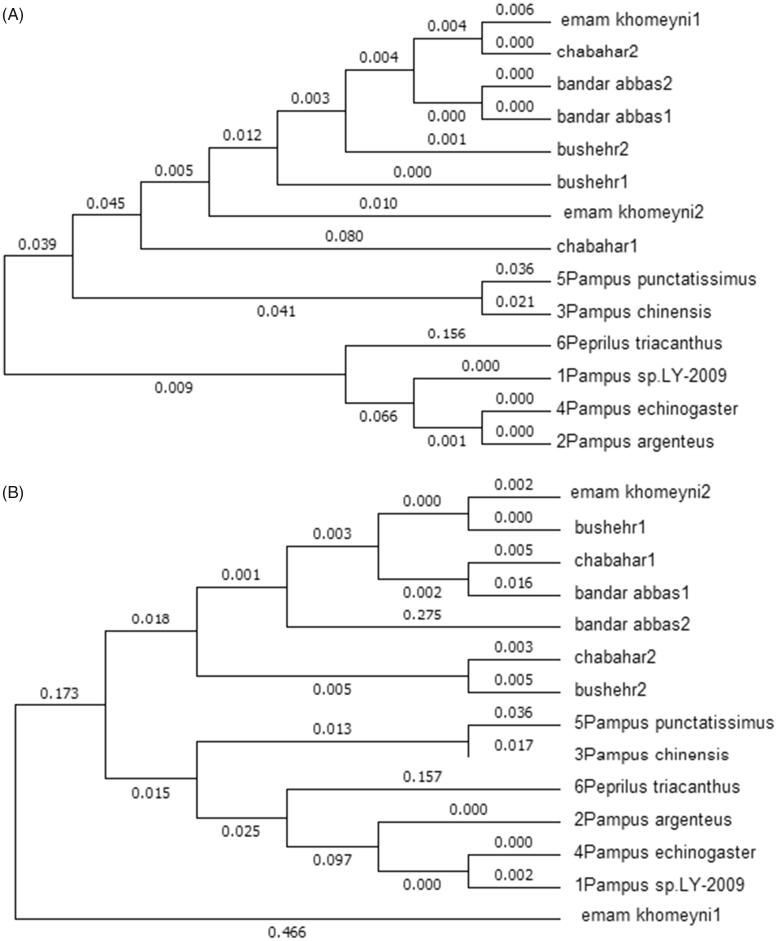
(A) Molecular phylogenetic analysis of cytochrome b gene by maximum-likelihood method. (B) Molecular phylogenetic analysis of D-loop gene by maximum-likelihood method.

Molecular phylogenetic analysis of cytochrome b gene by maximum-likelihood method was shown in Figure 2(A). All samples from Persian Gulf and Gulf of Oman are genetically more similar than samples of China Sea. Figure 2(B) was inferred molecular phylogenetic analysis of D-loop gene by maximum-likelihood method. Results are similar to cytochrome b excepting one sample (Emam Khomeyni1).

## Discussion

Phylogenetic trees interpret divergence between samples. Placement of samples in trees shows distinct groups and it gives us the opportunity to consider a common ancestor. More technically, the divergence represents the volume density of the outward flux of a vector field from an infinitesimal volume around a given point.

Sun et al. 2012 suggest that there is significant genetic divergence between silver pomfret fish in the area of the Arabian Sea, the Western Indian Ocean and the Sea of China. Analysis of the gene cytochrome b shows that genetic diversity observed in one population and there are fundamental differences between populations in different areas. Differences in habitat, migration, human activities and geographical separation are the factors that could be causing genetic divergence between sea animals. For example, Thailand and Burma population show little heterogeneity because of lack of boundaries separation. Vice versa geographical separation between the populations of the Arabian Sea and the South China Sea could be due to high genetic divergence. By comparing the similarities and differences in the nutrient supplies of silver pomfret populations in different regions, the similarity or difference between the populations is guessed. Ocean currents may also have different demographic structures which lead to a large divergence in demographic groups of silver pomfret.

In detail, with translocations in Persian Gulf to Gulf of Oman, similarity decreases slowly. Chabahar1 sample in phylogenetic tree of cytochrome b is phylogenetically near China Sea samples. The reason may be migration from ocean to gulf throughout the centuries and Chabahar is in Gulf of Oman and locationally near the Arabian Sea and Indian Ocean.

In D-loop region, results are similar to cytochrome b excepting one sample (Emam Khomeyni1). Divergence is deep and evolution is faster than cytochrome b gene. Altogether, D-loop in samples shows modification more than cytochrome b for the reason absence of protein product. Finally, it should be noted that it is needed more investigation that is determined relationship between samples in different areas and another species.
